# The Burden of Diabetes in Argentina

**DOI:** 10.5539/gjhs.v7n3p124

**Published:** 2014-11-17

**Authors:** Lorena González, Joaquín E. Caporale, Jorge F. Elgart, Juan J. Gagliardino

**Affiliations:** 1 CENEXA. Centro de Endocrinología Experimental y Aplicada (UNLP–CONICET), Centro Colaborador de OPS/OMS para Diabetes, Facultad de Ciencias Médicas (UNLP), La Plata, Argentina

**Keywords:** diabetes, DALY, YLD, YLL, disability, mortality, Argentina

## Abstract

**Objective:**

To measure the economic burden of diabetes in Argentina by age, gender and region for the year 2005, in disability-adjusted life years (DALYs).

**Methods:**

DALYs were estimated by the sum of years of life lost due to premature death (YLL) and years of life lived with disability (YLD).

**Results:**

In the population studied (20 to 85 years), the burden of diabetes without complications was 1.3 million DALYs, 85% of which were caused by disabilities. Whereas mortality rates (YLL) increased as a function of age, YLD showed the opposite relationship. Women had higher burden of disease values, represented by 51 and 61% of YLL and YLD, respectively, independently of age.

**Conclusions:**

Our results demonstrate that disabilities are a key component of diabetes burden; its regular and systematic estimation would allow to design effective prevention strategies, to assess the impact of their implementation and to optimize resource allocation based on objective evidence.

## 1. Introduction

Diabetes mellitus is a chronic and common disease and its prevalence is continuously rising worldwide. In Argentina, diabetes affects 9.6% of the adult population (Ferrante, Linetzky, Konfino, King, Virgolini, & Laspiur, 2011), being type 2 diabetes (T2DM) the most common (90%) clinical form (International Diabetes Federation [IDF], 2009).

Most people with T2DM develop acute (hyperosmolar coma and hypoglycemia events) or chronic (retinopathy, nephropathy, neuropathy, peripheral vascular disease, cardiovascular disease, cerebrovascular disease) complications. Although preventable, these complications are highly frequent, increasing the costs of care and decreasing the quality of life of people with the disease (Turner et al., 1998; UK Prospective Diabetes Study Group, 1998; Patel et al., 2008; Duckworth et al., 2009; Gaede et al., 2008; Slabaugh, Curtis, Clore, Fu, & Schuster, 2014; Caporale, Elgart, & Gagliardino, 2013; Elgart et al., 2014). The estimation of the negative impact of diabetes and its cause is necessary for health care managers to develop effective strategies to decrease such impact and improve the quality of life of people with the disease. Overall burden of disease can be estimated using a single and objective measure, the disability-adjusted life year (DALY) (Murray & Lopez, 1996), but unfortunately it is not widely used.

The concept of Global Burden of Disease (GBD) refers to a consistent set of estimates of morbidity and mortality using the DALY and the ICD-10 codes. The DALY is the sum of the years of life lost due to premature mortality (YLL) and the years of life lived with disabilities (YLD) within a given period of time, generally one year. In this context, DALYs measure the gap between the current health status (with or without a given disease) and an ideal situation where everyone reaches old age, free of disease and disability.

According to the last World Health Organization study of GBD, diabetes is the 19th leading cause of disease burden, with 19.7 million DALYs (Mathers, Fat, & Boerma, 2008). In Latin America, this scenario is even worse: diabetes is the 6th leading cause, accounting for 20% of DALYs. Unfortunately, and due to the type of data required to estimate indicators, DALYs are presented by region and not by country. Therefore, within-region homogeneity/heterogeneity characteristics are unknown. Despite this limitation, estimates by region have served as a guide to develop GBD recommendations for countries interested in studying the issue, as it occurred in Australia (Mathers, Vos, & Stevenson, 1999).

In the current report we estimate diabetes burden in Argentina for the year 2005 measured as DALYs and expressed as a function of age, gender and region.

## 2. Methods

### 2.1 DALYs Estimation

DALYs were obtained from the addition of two components: the years of life lost due to premature death (YLL), and the year of life lived with disabilities (YLD) (Murray, 1994; Fox-Rushby & Hanson, 2001). Both components were estimated using the procedure reported elsewhere (Murray & Lopez, 1996; Murray, 1994; Fox-Rushby & Hanson, 2001). Sensitivity of this measurement was evaluated with the procedures described by Drummond et al (Drummond, O’Brien, Stoddart, & Torrance, 1997) and Weinstein et al (Weinstein, Siegel, Gold, Kamlet, & Russell, 1996). This procedures reflected different choices for discounting rate and age weighting. The base case was reported without age-weighting (K=0) and undiscounted (r=0%).

To obtain population YLL and YLD, we also considered the number of deaths due to diabetes (YLL) and diabetes prevalence (YLD), expressed by age group and gender. Finally, both indicators were added (YLL + YLD) to obtain the DALYs lost during the year 2005.

### 2.2 Estimation of DALYs Ascribed to Diabetes

To estimate this parameter we used a slight modification of the methods developed by Mathers et al. (Mathers et al., 2008) and the Victorian Burden of Disease Study (State of Victoria, 2005). Accordingly, we estimated diabetes direct (due to diabetes *per se*) and indirect costs in relation to the two most frequent complications in both the general population and people with diabetes: acute myocardial infarction (AMI) and stroke. Diabetes chronic complications (blindness due to retinopathy, diabetic foot, neuropathy and amputations) were not included because of lack of local information. Indirect costs were estimated with population attributable fractions (PAF), diabetes prevalence by gender, age group and region (Ministerio de Salud de la Nación, 2005), and relative risks (Lanas et al., 2007; Almdal, Scharling, Jensen, & Vestergaard, 2004; Huxley, Barzi, & Woodward, 2006).

### 2.3 Years of Life Lost Due to Premature Death (YLL)

To estimate YLL, we considered the number of deaths caused by diabetes, AMI and stroke, and life expectancy by age group, gender and region. The number of deaths was obtained from the mortality database of Argentina for the year 2005 (Dirección de Estadísticas e Información de Salud [DEIS], 2006; DEIS, 2007), identifying ICD-10 codes for diabetes (E10, T1DM; E11, T2DM; E14, unspecified diabetes), AMI (I21) and stroke (I60, I61, I63, I64) by gender, age group and region. The inclusion of deaths due to E14 implies an assumption of deaths attributable to either T1DM or T2DM: we considered that deaths of people < 30 years of age were due to T1DM, while the remaining ones were attributable to T2DM. Finally, life expectancy by gender, age group and region was taken from mortality tables of the National Bureau of Statistics for the year 2005 (Instituto Nacional de Estadísticas y Censos [INDEC], 2005).

### 2.4 Year of Life Lived With Disabilities (YLD)

Their estimation included: 1) number of cases with each disease (diabetes prevalent cases and AMI/stroke incident cases) by gender, age group and region, 2) disability duration associated to each disease by age and gender, independently of region (Murray & Lopez, 1996), and 3) disability weight by disease.

### 2.5 Cases With Disease

Diabetes prevalence was obtained from the first National Survey of Risk Factors (Ferrante, & Virgolini, 2007; Ferrante et al., 2011), the QUALIDIAB records for Argentina (Gagliardino, De La Hera, & Siri, 2001a; Gagliardino, Hera, & Siri, 2001b), and the first results of the International Diabetes Management Practices Study (IDMPS) for our country (Chan et al., 2009; Ringborg et al., 2009): 90% of the cases were attributable to T2DM and the rest to T1DM or other. To avoid double-counting of direct and indirect costs, T2DM prevalence was adjusted by general population without complications (45.2% and 35.2% for T1DM and T2DM, respectively); this size represents the rate of people with diabetes without complications in Argentina, obtained from the IDMPS study (Chan et al., 2009; Ringborg et al., 2009).

Because of the lack of a precise and representative source of AMI and stroke incidence data, they were estimated using the formula: *Incidence rate=Mortality rate/Case fatality rate*, where *mortality rate* is the proportion of deaths in the general population by age, gender and region, and *case fatality rate* is the proportion of deaths on discharge in the total number of discharges also by age, gender and region. General population data were obtained from Vital Statistics for the year 2005 (DEIS, 2006) and discharge data were from the last available report on hospital discharges in people ≥ 35 years for the year 2005 (DEIS, 2007b).

### 2.6 Duration of Disabilities Due to Diabetes

Data by gender and age group were obtained from the burden of disease studies in Australia in 1996 and 2001 (Mathers, Vos, & Stevenson, 1999; State of Victoria, 2005), only considering the indicator for cases without associated complications (Murray, 1994).

Disabilities due to transient AMI (full capacity is recovered after 22 days [0.06 years]) were taken from the GBD Study (Murray & Lopez, 1996). In that study, stroke is defined as a disease with sequelae of different duration: i) mild disability for 6 months; ii) mild and permanent disability, iii) moderate and permanent disability, and (iv) acute and permanent disability. In this study, we included transient (0.5 year duration by age and gender) and permanent (obtained with software DISMOD II) disabilities.

### 2.7 Diabetes Disability Weight

It was obtained from the GBD study (Murray & Lopez, 1996) and Stouthard et al. results (Stouthard et al., 1997) for diabetes without complications (0.07), with AMI under treatment independently of gender and age group (0.395), and stroke, either transient (0.360) or permanent (equivalent to the mean of mild, moderate and severe disability: 0.360, 0.630 and 0.920, respectively), adjusted by prevalence by gender and age from the Australian study (Mathers, Vos, & Stevenson, 1999).

## 3. Results

In Argentina, diabetes burden without complications in the adult population (20–85 years of age) for the year 2005 represented 1.3 million DALYs (r=0; K=0), mainly due to disabilities (85%). YLL represented only 15% of total DALYs, demonstrating that disabilities are the key component of diabetes burden.

Table 1 shows the number of DALYs and their distribution at the different scenarios considered for parameters r and K, resulting in 89, 83 and 87% of DALYs lost for scenarios (r=0; K=1), (r=3%; K=0) and (r=3%; K=1), respectively.

**Table 1:** Years of healthy life lost due to diabetes

Scenario	YLL	%	YLD	%	DALYs
r=0; K=0	201,520	15	1,127,283	85	1,328,802
r=0; K=1	132,115	11	1,066,614	89	1,198,730
r=3%; K=0	153,892	17	760,223	83	914,115
r=3%; K=1	101,116	13	693,244	87	794,360

*Note*. YLL= years of life lost due to premature death, YLD= years of life lived with disability, DALY= disability adjusted life years, r= discount rate, K= age-weight, where 0 represent no age-weighting.

Table 2 shows DALYs due to diabetes (r=0, K=0) by gender and age group. It can be seen that the second most important number of YLD was recorded between 18 and 34 years of age (combined female and male data), a fact explained by the number of years these people must live with the disability rather than by the number of cases. On the other hand, the high rate of YLD recorded in the group of 50-64 years of age was due to the high diabetes prevalence measured at that age.

**Table 2:** DALYs lost due to diabetes by gender and age; rate per 100 people with diabetes

Age	YLL				YLD				DALYs		

	Men	Women	Total	%	Men	Women	Total	%	Men	Women	Total
18-34	2,353 (2)	3,020 (2)	5,373 (2)	2	103,859 (106)	227,115 (115)	330,974 (112)	98	106,212 (109)	230,135 (117)	336,347 (114)
35-49	8,342 (5)	7,416 (4)	15,758 (4)	5	111,615 (63)	170,388 (82)	282,003 (74)	95	119,957 (68)	177,804 (86)	297,761 (78)
50-64	39,912 (11)	30,789 (8)	70,701 (10)	16	169,324 (48)	211,930 (56)	381,254 (52)	84	209,236 (59)	242,719 (64)	451,955 (61)
≥65	48,311 (15)	61,375 (16)	109,687 (16)	45	52,075 (17)	80,977 (21)	133,052 (19)	55	100,386 (32)	142,353 (36)	242,739 (34)
Total	98,919 (10)	102,601 (9)	201,520 (10)	15	436,872 (46)	690,410 (60)	1,127,283 (54)	85	535,791 (56)	793,011 (69)	1,328,802 (63)

*Note*. YLL= years of life lost due to premature death, YLD= years of life lived with disability, DALY= disability adjusted life years. Between brackets, rate per 100 people with diabetes.

In order to better understand the YLL and YLD distribution by age groups, rates per 100 people with diabetes should be considered (values shown between parentheses). According to these rates, the association of mortality and disability with age was positive and negative, respectively. Likewise, this rate highlights the size of each component, being disability the most important one.

Finally, diabetes affected women more than men, with 51% and 61% of YLL and YLD, independently of age (data not shown).

Because of the relevance of the scale effect observed in Table 2 as rates, it is important to know the number of estimated cases by each condition evaluated, i.e., number of deaths due to diabetes, diabetes cases by type without complications, and AMI and stroke cases by age group. These data are presented in

Table 3, and they show that the higher number of cases were caused by T2DM and T1DM without complications, whereas AMI and different forms of stroke represented the lower number of cases. It can also be seen that our mortality data show a positive association between number of deaths and age (Table 3).

**Table 3:** Number of deaths due to diabetes, T1DM and T2DM without complications, and AMI and stroke by age

Number of cases	Age group (years)					

	18 - 34	35 - 49	50 - 64	≥ 65	Total	%
Deaths	439 (1)	1,934 (5)	7,965 (20)	29,413 (74)	39,751 (100)	5
T1DM without complications	23,917 (15)	30,459 (18)	62,332 (38)	48,100 (29)	164,808 (100)	22
T2DM without complications	74,503 (15)	94,880 (18)	194,167 (38)	149,832 (29)	513,383 (100)	68
AMI	0	2,223 (13)	5,555 (32)	9,506 (55)	17,284 (100)	2
Stroke	0	903 (6)	4,520 (31)	9,194 (63)	14,617 (100)	2
Total	98,859 (13)	130,399 (17)	274,540 (37)	246,045 (33)	749,843 (100)	100

*Note.* AMI: acute myocardial infarction. Between brackets, rate per 100 people with diabetes.

Table 4 shows the number of YLL and YLD due to each of the conditions studied by age group. It can be seen that a higher number of YLL was recorded at older age groups. Also, a clear inverse association between number of deaths and age was recorded in T1DM, whereas this association was positive in the case of T2DM.

**Table 4:** YLL and YLD by condition and age

	Condition	18 - 34 years	35 - 49 years	50 - 64 years	65 years or more	Total	%
YLL	T1DM	4,489	9,203	5,882	3,280	22,853	11
	T2DM	117	1,642	40,495	65,339	107,593	53
	AMI	219	1,919	11,279	19,094	32,510	16
	Stroke	548	2,994	13,046	21,974	38,563	19
	Total	5,373	15,758	70,701	109,687	201,520	100

YLD	T1DM without complications	80,434	68,554	91,897	30,426	271,311	24
	T2DM without complications	250,541	212,840	282,470	97,051	842,902	75
	AMI		3	26	36	65	0
	Stroke transitory		0	4	5	9	0
	Stroke permanent		605	6,857	5,534	12,995	1
	Total	330,974	282,003	381,254	133,052	1,127,283	100

	Total DALYs	336,347	297,761	451,955	242,739	1,328,802	

*Note.* YLL= years of life lost due to premature death, YLD= years of life lived with disability, DALY= disability adjusted life years, AMI= acute myocardial infarction.

In YLD, it is important to note the relative importance of T2DM without complications as compared to disability caused by other diseases.

## 4. Discussion

Following the Murray and López as well as the WHO reports (Murray & Lopez, 1996; Mathers et al., 2008; Mathers, Vos, & Stevenson, 1999), different authors have applied their methodology to evaluate disease burden in different countries. Our study is the first one that has used DALYs to estimate diabetes burden in Argentina for the year 2005; the precise estimation of this parameter would provide a useful tool to identify effective prevention strategies and to optimize resource allocation for their implementation.

According to the last WHO evaluation of GBD performed in 2004, diabetes was the 19th leading cause of disease burden, being responsible for 1.3% of total DALYs, and it was expected to rise to the 10^th^ position by the year 2030 (Mathers et al., 2008). More recently, Ikram et al. (2014) reported that in ethnic minorities of Netherlands, diabetes plays an important role in the disease burden, and that such burden will grow stronger than ethnic Dutch, resulting in a higher total disease burden in 2030. The authors encourage researchers to estimate the disease burden by ethnicity so that health priorities and preventive strategies can be settled to cope with the problem (Ikram, Kunst, Lamkaddem, & Stronks, 2014). Moreover, in their study, Kim et al. (2013) used DALYs to analyse insurance claim data while years of life lost (YLL) and years lost to disability (YLD) were measured in terms of incidence rate and number of deaths. They found that cerebrovascular disease was the leading contributor to the chronic disease burden followed by diabetes (E. Kim, Yoon, Jo, & H. Kim, 2013). Considering that diabetes is the 6^th^ leading cause of disease burden in the region of the Americas with 4.1% of total DALYs, the real increase would be higher than the estimated. This assumption is supported by the fact that in Latin America the first 10 leading causes were responsible for 35.5% of the total DALYs, and diabetes was the 8th leading cause, with 2.7% DALYs lost.

In their review of Latin American studies that measured DALYs, Gomez Dántes et al. (2011) stressed the key role of diabetes as a cause of DALYs in our Region. In fact, diabetes in Brazil was the first leading cause with 5.1% of the total DALYs lost (year 1998) (Jourdan, da Costa, Gonçalves, Mendes, Schramm, & Crisostomo, 1998); it was the 9th leading cause in Costa Rica (year 2005), with 4.6% of total DALYs (Ministerio de Salud de Costa Rica, 2005), while in Mexico in the period 2004-2007, the highest number of DALYs (5.5%) were attributable to diabetes (Lozano Ascencio, Frenk Mora, & González Block, 1996; Lozano, Gómez-Dantés, Franco, & Rodríguez, 2009). Although lower values were reported in Chile and Peru (1.9%) (Concha Barrientos, Aguilera Sanhueza, & Salas Vergara, 1996; Ministerio de Salud de Perú, 2006), in both countries diabetes was still among the first 10 causes responsible for DALYs.

Unfortunately, none of those studies calculated DALYs lost due to diabetes with the methodology used in our report and in the GBD study (Mathers et al., 2008), or considered indirect measuring methods (attributable burden of diabetes).

In Argentina only two reports have previously estimated diabetes burden, but with a different methodology from the one currently used. Rubinstein et al. (2010) measured the burden of cardiovascular diseases (AMI, unstable angina, stroke and others) attributable to different cardiovascular risk factors, including diabetes. In that report, 1.27 million DALYs due to cardiovascular disease were lost in 2005; from the total YLL and DALYs, around 14% of the total acute fatal and non-fatal events was due to diabetes (scenarios without social discount rate). Hypertension and hypercholesterolemia represented the highest rate of cardiovascular disease burden ascribed to risk factors. Unlike the present work, the authors considered diabetes as a modifiable risk factor and not as a disease; accordingly, diabetes was considered as a direct burden only through the incidence of associated cardiovascular events. All these results support the concept that cardiovascular disease plays a main role in the high DALY values induced associated to diabetes. The data reported by Murray et al. (2013) support this assumption. These authors used data of global burden of disease 2010 for 1990 and 2010 for the UK and 18 other comparator nations to analyze the trends and relative performance for mortality, causes of death, years of life lost (YLLs), years lived with disability (YLDs), disability-adjusted life-years (DALYs), and healthy life expectancy (HALE). Their study included 259 diseases and injuries and 67 risk factors or clusters of risk factors relevant to the UK. Their results showed that YLDs per person by age and sex have not changed substantially from 1990 to 2010 but age-specific mortality has been falling thus suggesting that the importance of chronic disability is rising. Among the major causes of YLDs in 2010 were cardiovascular risk factors being tobacco the leading one, followed by high blood and body-mass index. Diet and physical inactivity also accounted for UK DALYs in 2010. Additionally, these results opens new prevention avenues: promotion of healthy life style habits could help to decrease the global burden of disease associated to CVRF (Murray et al., 2013a). In this regard, it has been reported that glycaemic control is potentially cost effective at less than two times per capita gross development product while lipid control would produce a much smaller overall population benefit, but at an average cost effectiveness ratio similar to that for glycaemic control. Blood pressure control would produce an overall benefit between that for lipid control and that for glycaemic control (Salomon et al., 2012).

The Argentine National Ministry of Health, using the methodology developed by Murray and López, estimated the burden of disease for a large number of pathologies (Borruel, M., Mas, & Borruel, G., 2010). In this report, diabetes in the year 2005 was the seventh and sixth leading cause for men and women, respectively, being responsible for 128,576 DALYs. This figure represents less than 10% of the DALYs estimated in our study, a difference that could be attributed to either the different use of the same methodology or the inputs used to calculate DALYs.

In agreement with previously reported data (Mathers, Vos, & Stevenson, 1999; State of Victoria, 2005; Dantés et al., 2011; Rubinstein et al., 2010; Borruel et al., 2010; Gènova-Maleras, Álvarez-Martín, Catalá-López, Fernández de Larrea-Baz, & Morant-Ginestar, 2011; McKenna, Michaud, Murray, & Marks, 2005; Oliveira, Valente, Leite, Schramm, Azevedo, & Gadelha, 2009; Melse, Essink-Bot, Kramers, & Hoeymans, 2000; Murray et al., 2013b), in our study most DALYs were due to YLD and not to YLL. In this regard, Oliveira et al. (2009) reported that YLD represented around 72.5% of DALYs due to diabetes, while the remaining 27.5% was due to YLL. Likewise, our data showing that women and age group 45–69 years are the most highly affected are strongly supported by most data reported in the literature (Dantés et al., 2011; Gènova-Maleras et al., 2011; McKenna et al., 2005; Oliveira et al., 2009; Melse et al., 2000; Kominski, Simon, Ho, Luck, Lim, & Fielding, 2002).

One of the limitations of our study is the under reported number of deaths due to diabetes as primary or secondary cause (only less than 10% of cases are reported) (Oliveira et al., 2009); another limitation is that estimates do not consider different population groups since disease burden measured by DALYs differs with the different ethnic groups as shown by McKenna in the US (McKenna et al., 2005); similar differences could occur in our country. Finally, since we did not consider the sequelae assessed in the Australian Study (State of Victoria, 2005), our estimation must be considered conservative compared with that one.

Lack of information regarding the burden of diabetes and other chronic diseases is a serious obstacle to design health policies at different decision levels. In this regard, some provincial governments have tried to overcome this deficit through the implementation of the QUALIDIAB registry, a useful tool to evaluate medical quality of care and to characterize the clinical and metabolic condition of people with diabetes (Gagliardino et al., 2001a; Gagliardino et al., 2001b; Commendatore et al., 2013). The National Ministry of Health promotes the implementation of such registry in all Argentinean provinces as part of the Project for Strengthening the Basic Health Care Strategy. Such wide registry implementation would provide prevalence indicators of some of the above mentioned sequelae.

Despite the above mentioned limitations, our results provide evidence on the importance of disabilities induced by diabetes and of the feasibility of their estimation using the DALYs measurement. The data suggest that the regular and systematic assessment of DALYs would help to design prevention strategies and allocate resources according to the real demand; such measurement could be also useful to evaluate their potential beneficial impact.
